# Accountability and pediatric physician-researchers: are theoretical models compatible with Canadian lived experience?

**DOI:** 10.1186/1747-5341-6-15

**Published:** 2011-10-05

**Authors:** Christine Czoli, Michael Da Silva, Randi Zlotnik Shaul, Lori d'Agincourt-Canning, Christy Simpson, Katherine Boydell, Natalie Rashkovan, Sharon Vanin

**Affiliations:** 1 The Hospital for Sick Children, c/o Bioethics Department, The Hospital for Sick Children, 555 University Avenue, Toronto, ON M5G 1X8, Canada; 2 B.C. Children's & Women's Health Centre, 4500 Oak Street, Building K4-161, Vancouver, B.C. V6H 3N1, Canada; 3 Department of Bioethics, 5849 University Avenue, P.O. Box 15000, Halifax, NS, B3H 4H7, Canada; 4 The Hospital for Sick Children, c/o Child Health Evaluative Sciences, 555 University Avenue Toronto, ON M5G 1X8, Canada; 5 Ontario Hospital Association, 200 Front Street West, Suite 2800, Toronto, Ontario M5V 3L1, Canada

## Abstract

Physician-researchers are bound by professional obligations stemming from both the role of the physician and the role of the researcher. Currently, the dominant models for understanding the relationship between physician-researchers' clinical duties and research duties fit into three categories: the similarity position, the difference position and the middle ground. The law may be said to offer a fourth "model" that is independent from these three categories.

These models frame the expectations placed upon physician-researchers by colleagues, regulators, patients and research participants. This paper examines the extent to which the data from semi-structured interviews with 30 physician-researchers at three major pediatric hospitals in Canada reflect these traditional models. It seeks to determine the extent to which existing models align with the described lived experience of the pediatric physician-researchers interviewed.

Ultimately, we find that although some physician-researchers make references to something like the weak version of the similarity position, the pediatric-researchers interviewed in this study did not describe their dual roles in a way that tightly mirrors any of the existing theoretical frameworks. We thus conclude that either physician-researchers are in need of better training regarding the nature of the accountability relationships that flow from their dual roles or that models setting out these roles and relationships must be altered to better reflect what we can reasonably expect of physician-researchers in a real-world environment.

## Introduction

"I guess for me you know, in the morning I wake up and I say.... Am I more physician or more a researcher? Am I more a clinician or the other one?" [i]

Physician-researchers are bound by the ethical norms of both traditional medical practice and clinical research. Unfortunately, these norms can conflict. Traditional medical practice is primarily concerned with the best interests of individual patients, while clinical research is primarily concerned with creating generalizable knowledge. Practices best suited to fulfill one goal may undermine the other. Numerous attempts have been made and debated in the clinical and bioethics literature in order to establish both descriptive and prescriptive models for the relationship of these two roles. The models at the poles of the spectrum have been referred to as the 'similarity position' and the 'difference position' [1]. The similarity position posits that medical research is an extension of medical care, and thus generates similar professional obligations, while the difference position views medical care and research as having different aims and accordingly different obligations. A broad middle ground between these extremes serves as the third dominant mode of modeling physician-researchers' roles and obligations.

Missing from the current literature is research on the perspectives of physician-researchers themselves and whether any of the models used to describe their dual professional roles are compatible with their lived experiences. If the frameworks are not compatible with such lived experiences of physician-researchers, then it is possible that the expectations that regulators, employers and the public may have of physician-researchers will be unrealistic given that these expectations are based on the values of such frameworks; so too will the expectations that physician-researchers place on themselves. Given the importance of models for setting standards of professional education and practice [2], a lack of compatibility could also signal the need for greater attention to the expectations connected to the dual roles within medical and research training. If we accept the models as reasonable standards for the proper conduct of physician-researchers, then the limitations perceived by physician-researchers themselves may be a call to modify the models, alter and enhance professional pedagogy and change the parameters of practice.

This paper aims to contribute to the discussion by presenting findings from a qualitative research study entitled "Physician-researchers in paediatrics: A study on dual accountability", funded by the Canadian Institutes of Health Research and designed to explore the perspectives of physicians engaged in dual clinical-research roles. Little is known about the views, experiences and assumptions of physician-researchers and how these might inform treatment and research related decisions. The project comprised semi-structured interviews with thirty physician-researchers from three major pediatric hospitals across Canada. The intent of this paper is not to present the data, and/or provide a thematic analysis of results data (forthcoming elsewhere), but to examine whether physician-researchers' responses are in accord with the theoretical models discussed in the bioethics and research ethics literature and raise questions about how these dual roles are both framed and understood.

This paper begins with a brief overview of the methods used to gather input from physician-researchers followed by in-depth review of the models proposed to guide physician-researcher obligations. Next, interview data are summarized to illustrate the extent to which the views of physician-researchers interviewed regarding their dual roles either align or are in conflict with the proposed models. Lastly, the significance of a lack of clear alignment between the models considered and the perspectives of those interviewed is discussed.

## Methods

The work reported here was part of a larger qualitative study, informed by interpretive interactionism [3], which involves a commitment to trying to understand the meanings people make of their experiences in everyday life [4,5]. Epistemologically, it is based on the position that as people interact, they create their social realities and derive meanings about events in their lives [6]. Consistent with this approach, this study started from the perspective of clinicians who also conduct research. It was designed to yield insight into the subjective experiences of physician-researchers and how they interpret their dual roles.

The project comprised semi-structured interviews with 30 pediatric physician-researchers from three major pediatric hospitals across Canada. The study focused on the pediatric setting because research overlaps with treatment for many pediatric conditions due to their rarity; thus in tertiary environments, the vast majority of pediatric clinicians are engaged in these dual roles. The interviews were conducted in person (n = 29) at the participant's institution or by phone (n = 1) and covered a range of topics, including participants' descriptions of their professional roles related to clinical care and research, compatibility of their dual roles/responsibilities and how conflict is addressed. Interviews lasted from 30 minutes to 50 minutes in length. They were tape-recorded with prior consent of all participants and then transcribed verbatim. Participants consented to the publication of unidentifiable quotes.

Analysis involved an interpretive process and was guided by constant comparative techniques [3,7]. Initially, interview segments were grouped into preliminary categories based on interview questions (for example, role description-accountability, role separation-strategy, role conflicts, best practices, etc.). As more data were collected and coded with descriptive phrases or words, these categories were revised. Comparison of differences and similarities within and between categories and subcategories enabled further refinement, clarification of meanings and the development of conceptual themes. The data were managed using NVivo qualitative software program. This paper pays particular attention to how participants' descriptions of their dual roles fit with or contradict established models for guiding physician-researcher obligations.

### The Similarity Position

One model of the physician-researcher ↔ patient-subject relationship falls within a broad category referred to as the similarity position. The strong version of the similarity position posits that medical research is an extension of medical care, thereby denying any forms of tension between the two practices. The idea that medical progress is based on research provides the justification for the practice itself - "medical research involving human subjects may only be conducted if the importance of the objective outweighs the inherent risks and burdens to research subjects" [8]. If there is a conflict between the norms of medical care and the broader norms of research, it is expected that medical norms are given preference; medical research is thus a subset of medical practice and is primarily bound by medical practice norms. A view in which the obligations of clinical care trump research obligations is clearly outlined in the *Declaration of Helsinki *[8] and is also widely described in the medical and bioethics literature [9-11]. According to the Declaration, physician-researchers are primarily obligated to promote the health of their patients and act in their patients' best interests, even when engaged in research [8]. Thus, the traditional duties of practicing physicians are simply extended to the research realm.

The similarity position is not without its critics. As will be discussed in further detail below, some describe clinical care and research as having fundamentally distinct aims: clinical practice is concerned primarily with the good of the individual patient with whom the physician has a special relationship, while research is concerned primarily with the good of society. The main concern about the strong version of the similarity position is that it runs the risk of exacerbating the therapeutic misconception, viz., the mistaken belief that clinical research necessarily has therapeutic benefit, by failing to recognize the substantive differences between the aims and actions of clinical care and those of clinical research (let alone informing potential patient-participants of these differences) [12,13]. The dilemma can be articulated as follows: "How do we balance claims made upon us by immediate, particular individuals with whom we have special relationships, against competing claims of society as a whole, which has an interest in our participation in projects that promise widespread, future social benefit?" [14].

Benjamin Freedman addresses this potential problem in a manner consistent with the similarity position, albeit in a weaker form, i.e., via clinical equipoise [10]. For Freedman, clinical equipoise refers to a state of honest, professional uncertainty in the community of expert practitioners as to the preferred treatment among trial arms. Clinical equipoise retains the supremacy of clinical norms in physician-researcher practice, but assigns a moral locus to the community of expert clinicians. It emphasizes clinical evidence within the medical community rather than individual physicians' knowledge and preferences as the appropriate means of dealing with potential role conflict for the physician-researcher [15]. Clinical equipoise was developed as a method for resolving tensions in clinical research by framing research within the context of clinical care, mainly through reference to physicians' therapeutic obligation to patients [16,17]. Research is considered an acceptable alternative to standard treatment if, and only if, there is clear uncertainty as to whether research or standard treatment is best. Clinical equipoise is a core component of the *Declaration of Helsinki *[8] and the *Tri-Council Policy Statement *[18], which is the leading Canadian soft law on best practices in research. Freedman recognized that care and research are distinct enterprises, but nevertheless claimed that care duties trump those of research. This weaker similarity position does not view research as an extension of clinical care, but rather, perceives it as subordinate.

It is interesting to examine the extent to which physician-researchers place themselves within the similarity position model, i.e., as practicing physicians first and foremost or, in its stronger form, as practicing physicians only whose research is an extension of their practical work. Passages from our study interviews indicate this current of thought informs some physician-researchers' actions and professional self-identification. While study participants did not refer to the similarity position *per se*, their practice included some of its norms. When asked about the potential conflict between clinical and research roles, some physician-researchers responded as summarized by the following statement:

"It is also useful that I'm a clinician and [...] I can ask the right questions. So, I think that's synergistic and not a conflict."

Others indicated a physician's "duty is to provide the care to the patient and the family irrespective of their attitude towards clinical research", but then went on to discuss clinical research as "an additional benefit for clinical care" suggesting a strong relationship between them. Similarly, another physician-researcher observed different levels to practice:

"There's a conceptual level and there's the practical level. The conceptual level is I don't differentiate between my research and my clinical work so in other words the research questions that I ask in the studies that I conduct have direct implications to the clinical work. In fact, the research studies I've already conducted actually have changed the way I practiced."

Yet another explained how clinical research stems from clinical care:

"I don't feel much... conflict between my role as a clinician and the researcher role because my primary role is a clinician and out of that... I'm acting also scientifically but I don't have the scientific questions prior to the clinical ones."

Finally, another physician-researcher explained that:

"For the particular studies and things that I've been involved with up to now, there hasn't been a huge struggle for me. I haven't encountered that many situations where the two come in conflict. For me, the issue is always what I think is best for the patient and so, you know, if something has to give, it is the protocol."

While only one physician-researcher described clinical research and clinical care as "very much married together", the aforecited quotations are consistent with a weak version of the similarity position, as evidenced by a prioritization of clinical care norms and a perceived lack of conflict between the clinical and research roles.

### The Difference Position

At the other end of the spectrum, a second model of the physician-researcher ↔ patient-subject relationship grants preference to the practice of scientific research and is known as the difference position. The difference position's proponents suggest that the physician-researcher's relationship with the patient-subject must be modelled in a way that is reflective of the salient differences between clinical research and clinical care, giving preference to the former [19,20]. As with the similarity position, there are several different iterations of this model, but the overarching rubric is known as the "difference position".

The strongest claim made against extending the ethics of clinical care to clinical research concerns the different underlying goals of these two practices. Franklin Miller and Howard Brody argue that because of the differing goals of the two practices, the ethics governing those practices must also be different [19]. Proponents of the difference position claim that it should be adopted for clinical research so as to clarify the important differences between research and clinical care and, in turn, reduce physician-researchers' cognitive dissonance without fostering the therapeutic misconception [21]. They assert that it will help dispel the therapeutic misconception by clarifying the difference(s) between clinical research and clinical care. In other words, separating these two practices will help prevent a research subject from transferring "to the research setting the presumption that obtains in ordinary clinical treatment: that the physician will always act only with the patient's interests in mind" [13]. Recognizing the difference between clinical care and clinical research requires one to formulate a professional self-image in a manner consistent with the reality of the multiple norms incumbent upon him or her.

The difference position has, however, been heavily criticized. Trudo Lemmens and Paul Miller reveal that Miller and Brody seem to confer normative force to the observation that the goals of each practice are different, without providing an argument to account for it [22]. As well, Steinberg notes that "the dominant goals of clinical research and clinical care may differ; however, the existence of a goal does not suffice as its moral justification" [23]. In other words, the existence of a goal that is distinct from pursuing individual patients' best interests does not grant clinical investigators immunity from the ethical obligations of clinical care [23]. Steinberg's point is echoed by Kimmelman who points out that trials may embody multiple objectives: "I do not see a necessary relationship between the claim that research is *primarily *designed to answer scientific questions and the far stronger claim implicit in Franklin Miller's position - that, by its very nature, research is *exclusively *designed to serve the ends of others" [24].

Physician-researchers interviewed in our study did not stress the importance of clinical research over clinical care. They did, however, describe important differences between their aims. One participant not only recognized a difference between the two roles, but an inherent conflict between them:

"... I think there's absolutely conflict of interest in any situation where a clinician is a researcher as well."

Additional statements from the physician-researchers interviewed recognized both a difference between clinical care and clinical research as well as a possibility for ethical conflict. Interestingly, however, even those physician-researchers who expressed views in accordance with the difference position believed that in situations of role conflict, clinical norms should prevail. This suggests that strict adherence to the difference position may not accord with lived experience:

"If there's a dilemma you go with the ethical side.... The research has to take the back seat. I still have actually quite a lot of trouble with how the Tri-Council policies start and that is...something to the effect that everyone has a right to be in a research project. In other words, the way that it's painted is so positive."

Ultimately, this appears to be a middle ground position insofar as it recognizes a fundamental difference, but shares the similarity position's preference for clinical norms. Other physician-researchers in our study described strategies they used to keep their two roles separate. One participant stressed the importance of "...not trying to represent the two roles in the same point in time." Another described practice in the following way:

" I've been... trying to be very clear of which hat I'm wearing... I'm seeing you..., I'm your physician. We are not doing research today. And I will never go back and look at the data... for research. I really try not to be biased at all... I'll never ... mix the two."

Yet another physician-researcher stressed the importance of:

"...trying to separate [research] things from clinical care by having other people in to have discussions about things. ... I think you have to try and separate them."

Although several physician-researchers referred to a fundamental difference between the practices of medical care and research, and even devised strategies to help keep the two roles separate, we heard little that directly aligns with a strict difference position, perhaps indicating that a complete divorce between the two practices is uncomfortable for or undesired by physician-researchers.

### The Middle Ground

One could argue that both the similarity position and the difference position grant too much weight to one set of obligations, and thereby ignore the other set of obligations that make up the dual role of the physician-researcher. It is thus unsurprising that various accounts of the physician-researcher role have been put forth that assume a middle ground [17,25-28]. Each of these accounts attempts to characterize the nature of the physician-researcher ↔ patient-subject relationship in a way that lends credence to obligations that stem from both medicine and science. While we describe four different middle ground positions here, our physician-researchers only made reference to one.

Ezekiel Emanuel et al. present a model in which seven requirements must be met for research to be considered ethical [28]. The requirements present physician-researchers with ethical duties to respect the worth of every patient-subject, emphasizing an ideal of non-exploitation rather than therapeutic beneficence. Paul Litton and Franklin Miller support this model, arguing that individuals' autonomy should be respected by honouring individuals' choices to opt out of the patient role and the fact that they have reason to want the ways in which they are treated to depend on the choices they make [25].

Another approach to the middle position as described by Carl Coleman is based on the legal model of fiduciary relationships and rests on the question of "whether the researcher-subject relationship is sufficiently similar to a fiduciary one to warrant the application of a comparable legal approach" [17]. Coleman argues that the physician-researcher ↔ patient-subject relationship is characterized by the same type of vulnerability, trust and expectation of protection that underlies the relationship between fiduciaries and beneficiaries [17]. The purpose of ethical guidelines and the law should thus be to protect subjects from *excessive *deviations from their medical interests without demanding that researchers adhere to the same requirements they face in regular clinical care. This protection would be based on the concept of "fairness" or "reasonableness" *from a lay community point of view*, namely, an assessment of the trade-offs a reasonable person would be likely to accept [17]. In this way, Coleman argues that while researchers are not fiduciaries for their subjects, they are in a fiduciary-like relationship with them.

This idea is supported by Canadian law, in which fiduciary obligations in clinical care do carry over into the research setting (when conducting research on one's patient) [29-31]. Law does not, however, always frame how physician-researchers view their practice. At least one physician-researcher we interviewed was more pragmatic in understanding this position:

"It's not always standard of care. It's usually a judgmental issue which in your own clinical experience you may feel is detrimental to the care of the patient or in some case; it may be because you think something is not warranted to be done that is dictated by the protocol. For example, it may be an excessive amount of radiological investigations on the protocol for monitoring reasons which do not affect the care of the patient and so you may question whether doing those investigations is required."

A third middle ground model is put forth by Franklin Miller, Donald Rosenstein and Evan DeRenzo who believe that the dual role needs to be understood as a coherent moral identity that is "whole" and recognizes both sets of obligations, rather than oscillating between the two roles [26]. This holistic approach acknowledges that the challenging issues faced by physician-researchers are often responded to with the application of social regulatory measures, such as supervision by federal and institutional bodies. However, Franklin Miller et. al. note that these forms of regulation will not eliminate the tensions physician-researchers experience between patient care and scientific rigor; rather, the ethics of clinical research will continue to depend significantly on the integrity of investigators [26]. Physician-researchers will exhibit professional behaviour when they are able to recognize, understand and manage the inherent conflict of interests and obligations that come with their role.

Finally, Henry Richardson and Leah Belsky's partial entrustment model presents the patient-subject as partially entrusting his or her health to the physician-researcher, in contrast to the full entrustment embodied in the physician-patient relationship. Entrustment relationships, either full or partial, impose special duties of care on the entrusted person, due to the discretion given by and the vulnerability of the beneficiary. Richardson and Belsky compare their model to the concept of bailment in law, which involves an intermediate, limited entrustment [27]. In this context, a bailee is an individual who accepts custody of some particular good and is entrusted to look after it only in limited ways. The authors think this is a suitable model for the physician-researcher ↔ patient-subject relationship because the patient-subject authorizes the investigator to take custody of a valued item, i.e. their health, and because the investigator's superior position affords them the ability to judge how best to protect that item [27]. Analogizing from property law, Richardson and Belsky grant limited, but substantive, fiduciary obligations for the physician-researcher towards their patient-subjects.

### A Legal "Model"

Coleman and Richardson and Belsky both base their theoretical models on existent law, although the latter only analogizes from it rather than focusing on the actual state of the law. Importantly, there is a legal framework in place that establishes a final model worth considering here for its substantive force. Clinical care and clinical research are regulated separately, suggesting some acknowledgment of the difference between them. The former is regulated by statute in both the United States and Canada, while the latter is primarily regulated by soft law and the common law as informed by international standards. Best practices and standards for clinical practice are informed by documents including the *Nuremburg Code*, the *Declaration of Helsinki*, the Council for International Organizations of Medical Sciences (CIOMS)'s *International Ethical Guidelines for Biomedical Research Involving Human Subjects *and the American *Belmont Report*. Each of these documents reflects an acknowledgement of the difference(s) between clinical care and clinical research and contains provisions dedicated to minimizing the therapeutic misconception. Sections devoted to conflicts of interest are also acknowledged, although the conflict between the norms of clinical care and clinical research is rarely considered. Importantly, as mentioned above, the *Declaration of Helsinki *clearly states that clinical care duties are primary: "In medical research involving human subjects, the well-being of the individual research subject must take precedence over all other interests" [8].

Canadian research standards are also informed by a component of domestic policy, which serves the function of soft law. Canadian physician-researchers receiving research funding from the three major government granting agencies are bound by the *Tri-Council Policy Statement*. Past versions of this document largely followed the aforementioned models, but the *Tri-Council Policy Statement 2*, adopted in 2010, requires that physician-researchers disclose to research subjects/participants that they are dual role actors [18].

Canadian law recognizes that the roles of clinical research and clinical care are different and may potentially conflict. Any conflict must be brought to the attention of the potential patient-subject and resolved in accordance with the fiduciary duties incumbent upon physicians, even when they act in a research capacity. Unfortunately, physician-researchers have little guidance on how to reach this resolution since most regulatory bodies are silent on this issue. Furthermore, these fiduciary duties only apply when treating one's own patients.

## Conclusion

While many responses in the interview data could be viewed as being in line with a weaker version of the similarity position, the physician-researchers interviewed did not describe their dual roles in a way that *tightly *reflects any of the existing theoretical frameworks. This point can be interpreted in two possible ways: first, this lack of synergy could suggest that physician-researchers need to gain a better understanding of the models that regulators, employers, colleagues, patients and families may use in setting expectations and evaluating their actions; or second, this could highlight the need for models that better reflect the perspectives and experiences of physician-researchers themselves.

Models can be developed to be descriptive or prescriptive. If these models are considered descriptive, the pilot data from this study compels further research into the physician-researchers' understanding of their own dual accountability. If these models are prescriptive, these data call for greater education of physician-researchers to address the ethics and laws related to dual accountability.

Models or guidelines can be used to frame our expectations (and, indeed, best practices) of proper conduct of physician-researchers. When prevalent models do not cohere with the lived experiences of those to whom they apply, such models can distort expectations and foster ethical ambiguity relating to the role(s) and obligations of physician-researchers. Thus, we opine that there is a need for a model that is aligned with the lived experiences of physician-researchers, and at the same time maintains the expectations of the highest ethical conduct possible in that context. An emphasis on real-world practice abuts the same criticisms lobbied at the difference position of Franklin Miller and Brody, namely, Steinberg's suggestion that the fact of practice cannot serve as its moral justification. Simply put, the fact that something is done does not mean that it is the right thing to do. However, a morally justifiable model that is reflective of and responsive to current practice may provide greater insights into and guidance about, the genuine risks and benefits associated with the dual accountability of physician-researchers.

To conclude, greater appreciation of responsibilities and expectations appears to be needed on all fronts. Physician-researchers need to understand the expectations grounded in ethics and law that currently flow from both of their roles, and those who create these expectations need a better appreciation of the perspectives and insights of physician-researchers working in these dual roles.

## Endnotes

[i] This quotation is from one of thirty semi-structured interviews with physician-researchers at three major Canadian pediatric hospitals as part of the Canadian Institutes for Health Research-funded project "Dual Accountability of Pediatric Physician-Researchers." All unattributed quotations are from these interviews.

## Competing interests

The authors declare that they have no competing interests.

## Authors' contributions

CC performed a literature review, participated in the paper design, performed the initial draft manuscript and provided substantive reviews of the later drafts. MD participated in the paper design and redrafted the manuscript. LA, CS and KB helped design the research study and paper, contributed to analysis of the data, redrafting and critically reviewed the manuscript for intellectual content. NR conducted the interviews, participated in the transcription review and coding, data input (NVivo) and data analysis and theming. SV participated in paper design and critically reviewed the manuscript for intellectual content. RZS helped design the research study, contributed to analysis of the data, participated in paper design, redrafting and critically reviewed the manuscript for intellectual content; she also oversaw the research project. All authors read and approved the final manuscript.

## About the Authors

Christine Czoli HBSc: Research Assistant School of Nursing, Ryerson University and in the Emergency and Bioethics Departments, The Hospital for Sick Children.

Michael Da Silva BAH: JD candidate, University of Toronto, Research Assistant, Bioethics Department, The Hospital for Sick Children, Toronto.

Lori d'Agincourt-Canning MSc, M.A., Ph.D.: Assistant Clinical Professor, Pediatrics, UBC and

Director, Ethics Services, B.C. Children's and Women's Health Centre.

Christy Simpson Ph.D.: Head Department of Bioethics, Dalhousie University.

Katherine Boydell MHSc, PhD: Senior Scientist and Scientific Director of Qualitative Inquiry, Child Health Evaluative Sciences, The Hospital for Sick Children and Associate Professor, Department of Psychiatry, University of Toronto.

Natalie Rashkovan MHSc: Research Project Manager.

Sharon Vanin JD: Consultant, The Ontario Hospital Association.

Randi Zlotnik Shaul JD, LL.M., Ph.D.: Bioethicist, Associate Scientist Child Health Evaluative Sciences, The Hospital for Sick Children, Assistant Professor, Department of Paediatrics, University of Toronto.
